# Origin and differentiation of microglia

**DOI:** 10.3389/fncel.2013.00045

**Published:** 2013-04-17

**Authors:** Florent Ginhoux, Shawn Lim, Guillaume Hoeffel, Donovan Low, Tara Huber

**Affiliations:** ^1^Singapore Immunology Network, Agency for Science, Technology, and ResearchSingapore; ^2^Genome Institute Singapore, Agency for Science, Technology, and ResearchSingapore; ^3^Department of Biological Science, National University of SingaporeSingapore

**Keywords:** microglia, macrophage, central nervous system, origin, yolk sac

## Abstract

Microglia are the resident macrophage population of the central nervous system (CNS). Adequate microglial function is crucial for a healthy CNS. Microglia are not only the first immune sentinels of infection, contributing to both innate and adaptive immune responses locally, but are also involved in the maintenance of brain homeostasis. Emerging data are showing new and fundamental roles for microglia in the control of neuronal proliferation and differentiation, as well as in the formation of synaptic connections. While microglia have been studied for decades, a long history of experimental misinterpretation meant that their true origins remained debated. However, recent studies on microglial origin indicate that these cells in fact arise early during development from progenitors in the embryonic yolk sac (YS) that seed the brain rudiment and, remarkably, appear to persist there into adulthood. Here, we review the history of microglial cells and discuss the latest advances in our understanding of their origin, differentiation, and homeostasis, which provides new insights into their roles in health and disease.

## Introduction

Microglia are the resident mononuclear phagocytes of the central nervous system (CNS), belonging to the glial system of non-neuronal cells that support and protect neuronal functions. Microglia are broadly distributed throughout the brain and the spinal cord (Lawson et al., 1990), and account for 5–20% of the total glial cell population within the CNS parenchyma (Perry, 1998). Adequate and appropriate microglial function is crucial for the homeostasis of the CNS in both health and disease (Perry et al., 2010).

There are two main functional aspects of microglia: immune defense and CNS maintenance. As immune cells, they act as sentinels, detecting the first signs of pathogenic invasion or tissue damage in this delicate immune-privileged site that is actively protected by the brain blood barrier (Daneman, 2012). Under the inflammatory conditions of an active immune response however, microglia must also moderate the potential damage to the CNS and support tissue repair and remodeling. Perhaps unsurprisingly, dysregulated microglial activation and microglia-induced inflammation is observed in virtually all brain pathologies; emerging evidence suggests that microglia exert direct effects on neurons, contributing to disease progression (Perry et al., 2010; Kettenmann et al., 2011; Kingwell, 2012).

In recent years there has been an increasing appreciation of the importance of microglia for normal CNS function. In addition to their immune functions, emerging data are showing new and fundamental roles for microglia in the control of neuronal proliferation and differentiation as well as in the formation of synaptic connections (Graeber, 2010; Hughes, 2012). In the steady state, microglial cells constantly survey their local microenvironment, extending their motile processes to make transient contact with neuronal synapses, contributing to the modification and the elimination of synaptic structures (Tremblay et al., 2010). Microglia also contribute to the remodeling of post-natal neural circuits as they have been recently shown to play a role in synaptic pruning during post-natal development in mice (Paolicelli et al., 2011).

Thus, microglia occupy a central position in the defense and maintenance of the CNS and so are attracting interest as potential therapeutic targets in neurological disorders and recovery from brain injury. However, in order to exploit the abilities of the microglial population, we must first understand their origins and homeostasis before attempting to manipulate their functions. In this review we will present the latest advances in our knowledge on the origin of microglia, revisit early studies in light of recent developments and highlight some of the most relevant strategies to generate microglia for therapeutic approaches of neurological disorders.

## Historical perspectives on the nature of microglia

Defining the origin of microglia has been an elusive goal for generations of researchers and a longstanding issue of debate. Multiple schools of thought have emerged. The first description of the cells came from the work of Franz Nissl in the late nineteenth century, who described rod cells (“Stabchenzellen”) as reactive glial elements with migratory, phagocytic and proliferative potential. In the late nineteenth century, W. Ford Robertson introduced the term “mesoglia” to describe mesoderm-derived phagocytic elements in the nervous system that had origins distinct from those of neurons and neuroglia. Neuroglia were first described by Virchow, in 1856, who named them “nevernkitt” meaning nerve-glue, later translated as “neuroglia,” though in fact they corresponded to the macroglial population, which comprises astrocytes and oligodendrocytes (Rio-Hortega, 1939). While this idea had merit, in fact, Robertson's mesoglia similarly turned out to correspond mainly to oligodendrocytes. Santiago Ramon y Cajal later renamed the same cells the “third element of the nervous system” to further differentiate them from neurons and neuroglia, and stated that they were of probable mesodermal origin. This “third element” concept was refined further in 1919 by del Rio-Hortega, a student of Ramon y Cajal, who made the distinction between various cell types within the cells of the “third element” based on morphological and functional differences. Del Rio-Hortega introduced the term “microglial cell” to describe the non-neuronal, non-astrocytic third element as distinct from neurectodermal oligodendroglia or oligodendrocytes (Rio-Hortega, 1939) (For historic review see Rezaie and Male, 2002).

Although both W. Ford Robertson and Santiago Ramon y Cajal suspected a mesodermal origin of what were to become known as “microglial cells” (their mesoglia/third element of the nervous system), it was commonly held at the time that all glial cells were of neuro-ectodermal origin. Further dissecting the heterogeneity of the mesoglia, del Rio-Hortega was the first to introduce the term “microglia” to discriminate true mesodermal elements from oligodendrocytes, which were previously considered a component of the mesoglia. Del Rio-Hortega exploited silver staining techniques to describe the two types of cells as differing in origin, distribution, form, and function: the major population, called oligodendroglia, that lacked phagocytic activity, and the minor population of ramified resting cells. This minor population was then clearly defined as the “third element of the CNS” with a mesodermal origin, containing phagocytic corpuscles and with migratory and phagocytic activity (Rio-Hortega, 1932).

Despite del Rio-Hortega's seminal work, his theories were largely overlooked and have only recently come back to the forefront of scientific thinking (Rezaie and Male, 2002). At the time, there was much support for the belief that microglia shared a neuro-ectodermal origin with the other glial cells. Several studies supported this belief well into the twentieth century, including reports from Fujita who proposed a common, matrix-derived progenitor for microglia, astrocytes, and oligodendrocytes (Fujita and Kitamura, 1975). The work of Kitamura was similarly interpreted to indicate that microglia, as well as astrocytes, originated from neuro-ectodermal-derived glioblasts (Kitamura et al., 1984). As late as the 1990's, new studies continued to emerge that seemed to show a common origin of astrocytes and microglia; Hao reported that cultures of either murine embryonic neuro-epithelial cells or astrocytes could differentiate *in vitro* to give microglial-like cells, an idea which was supported by Fedoroff's work showing that clonal cultures of disaggregated neopallial cells from newborn mice gave rise to mixed microglial-astroglial cells (Hao et al., 1991; Fedoroff et al., 1997). In addition, data showing that donor bone marrow cells failed to contribute to the adult microglial population in either newborn (De Groot et al., 1992) or adult rodents (Matsumoto and Fujiwara, 1987) was interpreted to mean that the majority of microglial cells were of local neuro-ectodermal origin. However, this interpretation was soon updated in response to the finding that microglia (in contrast to other blood leucocyte populations) are highly radio-resistant. In 1993, Lassmann and co-workers were the first to demonstrate that resident microglia in rats are a very stable cell pool, in contrast to meningeal and perivascular macrophages, which in adult animals are only exceptionally replaced by circulating blood cells, even after recovery from severe brain inflammation (Lassmann et al., 1993). Such observations were later confirmed in mice by Priller, where the majority (85–95%) of microglial cells remained of host origin up to 15 weeks after bone marrow transplantation (Priller et al., 2001). The importance of the observation that newborn microglia are not replaced by donor bone marrow-derived cells (De Groot et al., 1992) will be discussed later, as it has the significant implication that the adult microglial population can be maintained solely by local radio-resistant precursors which are present in the brain prior to birth.

Other hypotheses on the origin of microglia included their derivation from the pericytes associated with blood vessels (Mori and Leblond, 1969; Baron and Gallego, 1972) or from the subependyma adjacent to the lateral ventricles (Lewis, 1968).

At the same time, a second school of thought was developing which paralleled del Rio-Hortega's original hypothesis. His conviction of the mesodermal origin of microglia was supported both by studies coupling light/electron microscopy and immunohistochemistry, which recognized typical morphological features of macrophages in the various stages of microglial development (Murabe and Sano, 1982), and by the demonstration that microglial cells reacted positively to antisera recognizing monocyte/macrophage antigens (Hume et al., 1983; Murabe and Sano, 1983). Despite the emerging evidence of the relationship of microglia to macrophages, other reports led to variable interpretations due to a lack of homology between monocytes and mature microglia in the expression of certain antigens, complicating the issue (Oehmichen et al., 1979; Wood et al., 1979). Nevertheless, the data showing phenotypic homologies between monocytes/macrophages and microglia were eventually validated by immunohistochemical studies that reported the specific expression of macrophage markers, including F4/80, Fc receptor and CD11b in mouse microglia (Perry et al., 1985), as well as FcGRI, and CD11b in their human counterparts (Akiyama and McGeer, 1990). Finally, a pivotal genetic study revealed that mice lacking PU.1, a crucial transcription factor for myeloid cells, were also devoid of microglia (McKercher et al., 1996; Beers et al., 2006). This unequivocally established the myeloid nature of microglia and simultaneously suggested that these cells might be ontogenetically related to macrophages.

## The origin(S) of murine microglia

Although there is a consensus about the myeloid origin of microglia, much controversy remains regarding the precise nature of microglial progenitors. Initial studies described the presence of microglial cells during early development, suggesting that microglia arise from embryonic progenitors. While del Rio-Hortega proposed that microglia originate from meningeal macrophages penetrating the brain during embryonic development, many authors including del Rio-Hortega himself, claimed that brain parenchymal microglia could also be derived from blood monocytes. Monocytes are indeed recruited to the neonatal and adult brain, in the latter case most often under inflammatory conditions, where they can differentiate into microglia-like cells. This knowledge long supported the prevailing viewpoint that circulating blood monocytes represent microglial progenitors, replacing those seeding the brain during embryonic development. In fact, until recently, the most consensual hypothesis was that embryonic and peri-natal hematopoietic waves of microglial recruitment and differentiation occurred in the CNS (Chan et al., 2007). However, we now know that the situation is somewhat different. Here, we will describe and discuss the recent advances in understanding of the origin of microglia, and will also revisit the data from earlier studies in light of these developments.

### Early development

In addition to having first described microglia, del Rio-Hortega also proposed that they might initially arise in the early stages of development from mesodermal cells of the pia mater, the innermost layer of the meninges (the membranes surrounding the brain and spinal cord). From his work on embryonic brains, he reported the “migration of embryonic corpuscles from the pia into the nerve centers” with morphological similarity to lymphocytes (Rio-Hortega, 1939). However, del Rio-Hortega also proposed that “microglia may eventually arise from other related elements, chiefly the blood mononuclears” based on the similarities in morphology and phagocytic activities of the microglia and monocytes (Rio-Hortega, 1939), thereby founding the “origin of microglia” controversy.

The immunohistochemical study conducted by Perry et al., more precisely described this phenomenon using macrophage markers such as F4/80. They concluded that as early as embryonic day 16 (E16) of development, macrophage-like cells that had extravasated into the brain parenchyma were localized in “hot spots,” from where they subsequently invaded the brain and differentiated through a series of transitional forms to finally become ramified microglia (Perry et al., 1985). Other studies later detected dispersed F4/80 expressing macrophages distributed within loose connective tissue surrounding the neuro-ectoderm in E12 rat embryos (Morris et al., 1991). Also in the rat, amoeboid microglial cells expressing monocytic markers are present as early as E12 in the neuro-epithelium (Wang et al., 1996). Interestingly, such embryonic cells were proposed to be microglial progenitors not only due to the similarities in phenotype and morphology, but because of their potent proliferative response to mitogenic stimulation *in vitro* (Alliot et al., 1991).

Similarly, in human fetuses, microglia-like cells with a range of morphologies can be detected from as early as 3 weeks of estimated gestational age (EGA) (Hutchins et al., 1990). However, it appears that maturation of the microglial compartment is ongoing throughout the majority of gestation: colonization of the spinal cord begins at around 9 weeks, the major influx and distribution of microglia commences at about 16 weeks, and ramified microglial forms take up to 22 weeks to become widely distributed within the intermediate zone (Rezaie and Male, 1999; Rezaie et al., 2005). It is only close to term, at 35 weeks, that well-differentiated microglial populations can be detected (Esiri et al., 1991) (for review Rezaie, 2003 and Verney et al., 2010).

Altogether, these seminal studies strongly suggested that microglia derive from embryonic hematopoietic precursors that seed the CNS prior to birth and, more importantly, before the onset of bone marrow hematopoiesis. However, the exact tissue origin and developmental cell lineage of precursors that migrate to the CNS to give rise to the “first” endogenous wave of microglia remained unknown and a topic of debate until recently.

### Mouse and human embryonic hematopoiesis

A major challenge in defining the embryonic microglial precursor was the complication of the dual source of blood cell formation during embryogenesis. Two major hematopoietic sites contribute to this process: the extra-embryonic yolk sac (YS) and the fetal liver (Tavian and Peault, 2005; Orkin and Zon, 2008). In mice, primitive hematopoiesis initiates in the YS around E7.0, shortly after the onset of gastrulation, leading mainly to the production of erythrocytes and macrophages (Moore and Metcalf, 1970; Palis et al., 1999; Bertrand et al., 2005). Primitive macrophages first appear in the blood islands of the mouse YS on the ninth day of gestation and their pattern of differentiation is unique in the sense that they do not go through a monocytic intermediate stage, as seen in adult macrophages (Takahashi et al., 1989). YS-derived primitive macrophages will spread into the embryo proper through the blood after the circulatory system has been fully established (from E8.5 to E10) (McGrath et al., 2003) and migrate to various tissues, including the brain. Once in the tissues, they differentiate into so-called “fetal macrophage populations” even before the onset of monocyte production by the fetal liver (Naito et al., 1990). These fetal macrophages have high proliferative potential, not only in the YS where they are produced but also in the tissues that they colonize (Takahashi et al., 1989; Sorokin et al., 1992; Naito et al., 1996; Lichanska and Hume, 2000). After E8.5, with the determination of the intra-embryonic mesoderm toward the hematopoietic lineage, a new wave of hematopoietic progenitors is generated within the embryo proper, first in the para-aortic splanchnopleura (P-Sp) region and then in the aorta, gonads, and mesonephros (AGM) region (Godin et al., 1993; Medvinsky et al., 1993). The hematopoietic stem cells generated within the AGM will lead to the establishment of definitive hematopoiesis (Orkin and Zon, 2008). Around E10.5, YS- and AGM-derived hematopoietic progenitors colonize the fetal liver (Kumaravelu et al., 2002), which serves as the major hematopoietic organ after E11.5, generating all hematopoietic lineages, including monocytes (Naito et al., 1990). A recent study highlighted further differences between primitive and definitive hematopoiesis, showing that the latter relies on the transcription factor Myb, while YS-derived macrophages are Myb-independent. This further underlines the fact that YS-derived macrophages constitute an independent lineage, distinct from the progeny of definitive hematopoietic stem cells (Schulz et al., 2012).

Human hematopoiesis also begins in the YS around day 19 of the EGA, and YS-derived stem cells are similarly limited/committed to myelo-erythroid development. Hematopoiesis then moves transitorily to the fetal liver around 4–5 weeks EGA, before being definitively established in the BM approximately at 10.5 weeks EGA (Tavian and Peault, 2005).

### The yolk sac hypothesis of microglial origin

Ashwell was the first to report the presence of round and amoeboid microglial cells in the fetal mouse cerebellum (Ashwell, 1990) and then in rat forebrain (Ashwell and Waite, 1991) as early as E11.0. Sorokin soon after detected macrophage-like cells and their precursors in blood vessels and the embryonic mesenchyme in rat embryos from 10.5, and noted that the developing brain was the first organ to be colonized (Sorokin et al., 1992). Interestingly, cells with the capacity to differentiate into microglia-like cells *in vitro* (expressing Mac-1, Mac-3, F4/80 and Fc antigens, with a macrophage-like morphology and ultrastructure) can be detected in the developing neuro-epithelium at days E8.5/E9.0, suggesting that in mice, the earliest developmental stage at which seeding of cells with myeloid features occurs in the brain is at E8.5/E9.0 (Alliot et al., 1991). Later reports confirmed the presence at similar stages of amoeboid cells expressing macrophage (Alliot et al., 1999; Ginhoux et al., 2010) and microglial markers (Chan et al., 2007; Mizutani et al., 2012) in both the cephalic mesenchyme and the neuro-epithelium, in accordance with the idea that the YS contributes to microglial genesis.

However, the evidence for a YS origin of such microglial progenitors was, at first, mixed. Initially, data from one of the aforementioned *in vitro* studies were interpreted to support the hypothesis that these macrophage-like cells that will give rise to microglia originated from the neuro-ectoderm (Hao et al., 1991). Takashi and Naito drew a different conclusion after they described the first emergence of immature macrophages within blood islands of embryonic YS at fetal day 9 in both mouse (Takahashi et al., 1989) and rat (Takahashi and Naito, 1993). Following the establishment of fetal blood circulation, these cells colonize the embryonic tissues, including the brain rudiment. By [3H]-thymidine autoradiography, YS macrophages were shown to possess high proliferative potential, which suggested that these fetal macrophages were in fact primitive macrophages from the YS (Takahashi and Naito, 1993). Alliot also clearly and convincingly proposed that such cells were true microglial progenitors of YS origin, as, at that stage, the YS is the only hematopoietic site in the embryo. This group then conclusively documented the presence of potential microglial progenitors in the YS and then the brain rudiment, with their numbers increasing dramatically from E9.0/E9.5 until around 2 weeks after birth (Alliot et al., 1999).

A similar pattern of events is likely observed in humans, where, from 4.5 weeks gestation, amoeboid microglial cells (characterized by the expression of Iba1, CD68, CD45, and MHC-II) enter the cerebral wall from the ventricular lumen and the leptomeninges (Rezaie et al., 2005; Monier et al., 2007). In the YS and mesenchyme at 4–6 weeks after fertilization, two populations of cells with a dendritic morphology could be distinguished: a majority that expressed monocyte/macrophage-associated markers but no detectable HLA-DR antigen, while the minority constitutively expressed MHC class II (HLA-DR and -DP) but no monocyte/macrophage-associated markers (Janossy et al., 1986). The emergence of this heterogeneity preceded the formation of both thymus and bone marrow, suggesting the independent development of these macrophage populations (for review Verney et al., 2010).

Interestingly, the YS derivation of microglia appears to be conserved across diverse species as shown in the zebrafish (Herbomel et al., 2001), and in avians (Cuadros and Navascues, 1998). However, what differs in mice is the requirement for a functional blood circulation for the spreading of YS macrophages in the embryo proper. In zebrafish, this colonization appears independent of the blood circulation as YS macrophages first directly invade the whole cephalic mesenchyme, and from there invade epithelial tissues including the brain, while other macrophages enter the blood circulation (Herbomel et al., 1999). Similarly, using chick-quail blood chimeras, Kurz showed that in avians, YS macrophages do not penetrate through the wall of embryonic CNS vessels, but rather from the pial surface (Kurz et al., 2001). In contrast, in mouse embryos, there is a clear requirement for the circulatory system as E9.5–E10.5 *Ncx-1^−/−^* embryos, which lack a heartbeat and therefore have no functional blood circulation due to a defect in the sodium calcium exchanger 1 (Koushik et al., 2001), also lack microglial progenitors (Ginhoux et al., 2010), as well as other fetal macrophages. However, this defect does not affect YS hematopoiesis as *Ncx-1^−/−^* embryos have similar numbers of YS macrophages as their control, normal phenotype littermates (Ginhoux et al., 2010). Whether murine YS macrophages enter via the blood circulation directly in the brain parenchyma or first enter in the cephalic mesenchyme and then migrate to the neuro-epithelium (Chan et al., 2007) remains to be clearly defined.

**Figure 1:**
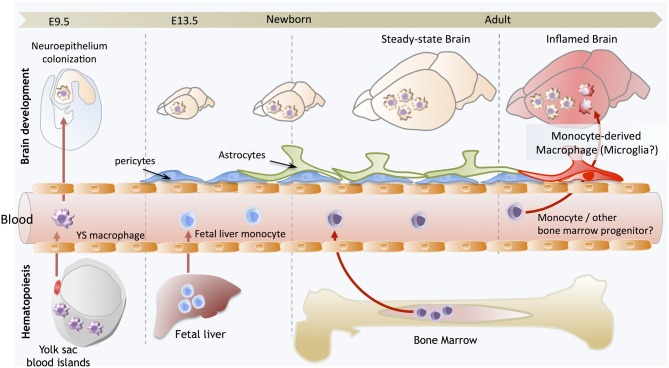
**Brain development and microglial homeostasis.** Primitive macrophages exit the yolk sac blood islands at the onset of circulation and colonize the neuroepithelium from E9.5 to give rise to microglia. The blood brain barrier starts to form from E13.5 and may isolate the developing brain from the contribution of fetal liver hematopoiesis. Embryonic microglia expand and colonize the whole CNS until adulthood. Importantly, in steady state conditions, embryonically-derived microglia will maintain themselves until adulthood, via local proliferation during late gestation and post-natal development as well as in the injured adult brain in reaction to inflammation. Nevertheless, during certain inflammatory conditions found for example after bone marrow transplantation, the recruitment of monocytes or other bone marrow-derived progenitors can supplement the microglial population to some extent. However, we do not understand yet whether these cells persist and become integrated in the microglial network, or are a temporary addition to the endogenous population.

The overall conclusion of these studies in rodents, humans, and other species is that microglia derive from the YS macrophages that seed the brain rudiment during early fetal development (Figure 1). However, these reports could not exclude the possibility that others progenitors could supersede the YS contribution. In fact, some data that will be discussed below, continued to emerge that suggested a requirement for the contribution of blood-borne cells to both generate the post-natal microglial compartment, and to maintain it into adulthood.

### The early post-natal contribution of monocytes to the microglial population

Shortly after birth in rodents, the microglial population expands dramatically (Alliot et al., 1999; Tambuyzer et al., 2009), leading to the suggestion that the proliferation of embryonic microglial cells alone could not account for the steep rise in numbers and that there must be a fresh influx of cells from another compartment. As initially suggested by del Rio-Hortega, blood monocytes were believed to invade the CNS in the perinatal period and give rise to microglia, replacing the embryonic microglial cells.

There was support for this belief from several studies, notably an early report where round, amoeboid, phagocytic cells were seen in rat corpus callosum during the first few days of life and then disappeared coincident with the appearance of ramified microglia. These cells were typical macrophages, but some displayed features of monocytes, while others appeared to be transitional between the two types. The authors of this study concluded that circulating monocytes enter the developing brain to assume the form of ameboid microglia that subsequently evolved to become ramified microglia (Ling, 1976). Subsequent studies gave neonatal rats an intra-peritoneal pulse of [3H]-thymidine to allow tracking of labeled blood cells by autoradiography. Labeled immature amoeboid cells were detected in the corpus callosum few hours after administration, while the majority of newly-ramified microglia were labeled one week later. These observations implied that labeled microglial cells must therefore have come from the transformation of immature amoeboid cells that acquired the tracer earlier (Imamoto and Leblond, 1978). This possibility was further tested by injecting a suspension of carbon particles into the circulation of rats of various ages to enable tracing of carbon-labeled monocytes, or by direct adoptive transfer of carbon-labeled monocytes. Later on, carbon particles were sequentially found in amoeboid cells of the corpus callosum and then on ramified microglial cells, suggesting again that blood monocytes, after ingesting carbon particles in the circulation or after transfer, migrated to the corpus callosum and differentiated into microglial cells via an amoeboid stage (Ling, 1979; Ling et al., 1980; Leong and Ling, 1992). However, while such data suggest that circulating blood monocytes can enter the CNS right after birth, perhaps in a specific site, it is important to note that such studies were rather qualitative and did not clearly address the exact relative contributions of post-natal monocytes versus embryonic progenitors to adult microglial homeostasis. In fact, the authors had themselves clearly recognized that such events were infrequent (Ling et al., 1980).

Nevertheless, later studies employing the PU.1 knockout (KO) mouse model, that lacks embryonic microglia, demonstrated the capacity of bone marrow-derived cells to contribute to the post-natal microglial population. In this study, neonates received wild-type bone marrow transplants within 24 h of birth, which resulted in *de novo* generation of the full microglial compartment (Beers et al., 2006). Therefore, it must be concluded that, at least under exceptional circumstances such as in the PU.1 KO mouse where endogenous embryonic microglia are completely absent, some bone marrow-derived cells have the capacity to infiltrate the CNS and assume the morphology and phagocytic capacity of microglia.

### A contribution of monocytes to the adult microglial population in the steady state?

Following the observations that monocytes might be able to contribute to the microglial population immediately after birth, it became implicitly accepted that they could also do so in adults. The idea that monocytes, or any bone marrow-derived cells, might then be able to be engineered and used as a delivery system into the CNS for therapies, the “Trojan Horse” theory, motivated investigators to discover the underlying mechanisms. The main hypothesis became that embryonic microglia disappear and are replaced by post-natal bone marrow-derived cells.

That is not to say that there were not data supporting the hypothesis of a role of monocytes in maintaining the adult microglial population: a seminal study employing [3H]-thymidine incorporation and autoradiography in normal adult mice concluded that cells can be recruited from the circulating monocyte pool through an intact blood-brain barrier (BBB) and rapidly differentiate into resident microglia (Lawson et al., 1992). However, the authors also noted that the resident microglia were proliferating, which suggested that the microglial population might maintain itself through either mechanism, or perhaps both. Other data supporting the idea that adult bone marrow-derived cells can give rise to microglia included the observation that following total bone marrow transplantation, some donor hematopoietic cells differentiated into microglia within the brains of adult mice (Eglitis and Mezey, 1997; Mezey et al., 2000; Simard and Rivest, 2004).

However, these results were in slight disagreement with a previous, and importantly, more quantitative, study which showed that the majority of microglial cells remained of host origin up to 15 weeks after bone marrow transplantation in mice (Priller et al., 2001). Similar results were also initially reported in rats as several investigators concluded that there was little or no contribution of bone marrow-derived cells to the adult microglial pool (Matsumoto and Fujiwara, 1987; Lassmann et al., 1993). In addition, it was shown that while microglia are not bone marrow-derived in adults, the closely-associated meningeal and perivascular macrophages are, perhaps going some way to explaining the confusion. Schelper and Adrian bluntly concluded that “monocytes become macrophages; they do not become microglia,” in this case, following CNS lesions (Schelper and Adrian, 1986), while Hickey and Kimura showed that the stable pool of resident microglia is only exceptionally supplemented by hematopoietic cells, even after recovery from severe brain inflammation (Hickey and Kimura, 1988). Similarly, Vallieres reported that many of these cells were in fact perivascular macrophages and that newly-formed parenchymal microglia were found in significant numbers only in the cerebellum and at injury sites (Vallieres and Sawchenko, 2003). Importantly, in humans, taking advantage of sex-mismatched donor bone marrow transplant (male into female) where Y-chromosome specific *in situ* hybridization can be performed to follow the origin of cells, similar results were obtained. The only donor male cells detected corresponded to mononuclear leucocytes within the vessel lumen and infiltrating the perivascular space and parenchyma, and perivascular cells (Unger et al., 1993). In fact, the observation that bone marrow-derived microglia were only found in notable amounts under certain conditions highlighted some significant shortfalls of the earlier studies: while it was successfully shown that monocytes could differentiate into cells that resembled microglia, few had quantified the effect or attempted to define the phenomenon in space and time, or to monitor the persistence of the bone marrow-derived microglia.

### A contribution of monocytes to the adult microglial population during inflammation?

What became clear was that although the monocyte-to-microglia path may exist in adult brain, it is unlikely to be a significant source for maintaining the microglial population, although this might change during CNS inflammation or disease (Vallieres and Sawchenko, 2003; Ladeby et al., 2005; Mildner et al., 2007). In fact, in response to CNS inflammation and damage, an increase in microglial number is often observed, a phenomenon called reactive microgliosis, which has become a hallmark of many CNS pathologies. However, it remained to be elucidated whether such increases in number rely on local expansion of mature microglia or are achieved by recruitment of blood precursors such as monocytes.

Two recent studies clarified the relative contribution of blood monocytes to microglia in experimental models of CNS pathologies. Both revealed that the irradiation regimen used to prepare recipient animals for bone marrow transplants is necessary for the recruitment and differentiation of monocytes into microglia (Ajami et al., 2007; Mildner et al., 2007). Mildner showed that recipient mice in which the CNS was shielded to protect from the irradiation and its associated inflammation, which induces the release of pro-inflammatory cytokines and chemokines, did not experience a significant invasion of bone marrow-derived cells into the brain, in contrast to the unshielded mice (Mildner et al., 2007). Beyond the irradiation issue, these data also suggest that microglial engraftment from the blood requires pre-conditioning of the CNS that likely disrupts the BBB. Additional clarity came from experiments in parabiotic mice, which have undergone surgery to physically link their circulatory systems, providing a more physiological means to study the turnover of hematopoietic cells for prolonged periods without the need for irradiation (Ajami et al., 2007). Ajami used such mice to show that in contrast to what was observed in irradiated and transplanted mice, there was no microglial progenitor recruitment from the circulation in either denervation or CNS neurodegenerative disease, despite the fact that the mixing of leucocyte populations can reach up to 50% in the blood of both parabionts. In agreement with their findings, we found no contribution of bone marrow-derived cells to CNS microglia up to 12 months after parabiosis (Ginhoux et al., 2010). Such data suggest that maintenance and local expansion of microglia are solely dependent on the self-renewal of CNS-resident cells in these models.

Interestingly, with this parabiotic model, in the context of irradiation of one parabiont, no further contribution from the other parabiont was detected in contradiction with the results of Mildner. However, Ajami further clarified that although irradiation is required for donor cells to engraft, it is not sufficient; another important, but overlooked, requirement is the artificial introduction of a critical number of bone marrow cells into the blood circulation (where they are not normally found) in conjunction with the inflammation of BBB caused by irradiation, a situation found only upon lethal total bone marrow transplantation (Diserbo et al., 2002; Li et al., 2004; Linard et al., 2004). More recently, the same group used a similar approach combining parabiosis and myelo-ablation to show that recruited monocytes do not persist and therefore do not contribute to the resident microglial pool. However, recruited monocytes contribute to the severity of disease in multiple sclerosis and the experimental autoimmune encephalitis mouse model (Ajami et al., 2011). Similarly, in transgenic mouse models of Alzheimer's disease, irradiation was shown to condition the brain for engraftment of myeloid cells, a phenomenon that does not occur normally during disease progression (Mildner et al., 2011). Interestingly, in this study, perivascular macrophages, rather than microglia and monocytes, modulated β-amyloid deposition in the brains of AD transgenic mice by clearing Aβ in a CCR2-dependent fashion (Mildner et al., 2011). This highlights the distinct and non-redundant roles of microglia, monocyte, and perivascular macrophages in acute injury and autoimmune inflammation (Jung and Schwartz, 2012). Finally, Capotondo recently clarified that the conditioning regimen also contributes to the ablation of endogenous microglia, thereby allowing the local proliferation of invading blood cells (Capotondo et al., 2012). In conclusion, parabiotic mice provided, for the first time, unequivocal evidence that the microglial population during the steady state is able to maintain itself throughout adult life by local renewal, independent of circulating precursors in steady state. Conversely, in transplant models, which are perhaps not so much reflections of normal physiology, a fraction of microglia can arise from adult bone marrow.

As discussed before, we know that adult bone marrow cells can also enter into the CNS and differentiate into microglia in exceptional circumstances: when the endogenous microglial niche is completely vacant, such as in the PU.1 KO (Beers et al., 2006), or experimentally depleted, for example using Gancyclovir in a mouse model expressing the thymidine kinase under the CD11b promoter (Varvel et al., 2012). Importantly, microglial repopulation in the latter study did not require any conditioning regimen such as irradiation, as the microglial pool reconstituted itself after cessation of the Gancyclovir treatment. However, the effect of Gancyclovir on the permeability of the BBB was not evaluated in this model and we do not know if the bone marrow progenitors require additional “help” in order to cross the BBB, perhaps through the presence of as yet undefined inflammatory mediators. In addition, in their experimental setting, the authors could not formally track the origin of the cells that repopulate the microglia and therefore were unable to exclude local repopulation from non-depleted microglial cells. Nevertheless, the fact that adult bone marrow cells can give rise to microglia in the context of hematopoietic cell transplantation with a conditioning regimen open the door for invaluable therapeutic strategies for the correction of CNS conditions in which defects of microglia are implicated. For example, bone marrow transplantation of mouse models for metachromatic leukodystrophy (Biffi et al., 2004), the obsessive compulsive disorder trichotillomania (Chen et al., 2010), and Rett syndrome (Derecki et al., 2012) has been shown to ameliorate disease symptoms.

In conclusion, in these transplant models, which are perhaps not so much reflections of normal physiology, a fraction of microglia are of bone marrow origin. However, during the steady state, monocytes or other bone marrow-derived cells do not enter the CNS and do not significantly contribute to the microglial population.

### Evidence for the persistence of the embryonic wave of microglia

What remained unclear in the field, however, was the relative contribution of embryonic and post-natal hematopoietic progenitors to the steady-state microglial population in adults: are the embryonic microglia responsible for maintaining the adult pool or do embryonic and adult microglia in fact have different origins? One of the studies already discussed, from De Groot, had implied that embryonic microglia were the sole contributors to the adult microglial pool. This study observed that donor bone marrow cells failed to contribute to the adult microglial population in a model of newborn transplantation, and concluded therefore that the adult microglial population was totally independent of post-natal bone marrow-derived circulating precursors from birth onward (De Groot et al., 1992). Recently, we revisited their experiments with a more quantitative aim and found that while most circulating leucocytes were of donor origin, the majority of microglia remained of host origin for more than 3 months after transplantation, confirming that post-natal hematopoietic precursors, including monocytes, likely do not contribute to the adult microglial population (Ginhoux et al., 2010).

We also employed a more advanced technique of YS progenitor fate mapping to definitively answer this question. Our fate mapping mouse model expresses a fluorescent protein (eYFP) exclusively in YS progenitors and their progeny, which include YS macrophages. Briefly, this mouse model expresses a tamoxifen-activated *MER-Cre-MER* recombinase gene under the control of one of the endogenous promoters of the runt-related transcription factor 1 (Runx1) locus (Samokhvalov et al., 2007). When crossed with a Cre-reporter mouse strain, recombination can be induced in embryos by a single injection of 4-Hydroxytamoxifen (4′OHT) into pregnant females. Active recombination in these knock-in mice occurs in a short time frame that does not exceed 24 h post-injection and leads to irreversible expression of eYFP in Runx1^+^ cells and their progeny (Samokhvalov et al., 2007).

Although both YS and fetal liver hematopoietic progenitors express Runx1, YS progenitors are the only cells present at E7.5 and so injection of tamoxifen at E7.5 will therefore allow the specific and irreversible tagging of YS progenitors and their progeny but not of fetal liver-derived progeny. In contrast, injection of tamoxifen at later time points (from E8.5) will favor the tagging of AGM-derived hematopoietic progenitors and not the YS progenitors (North et al., 1999; Samokhvalov et al., 2007). We can use this model to accurately ask about the origins of different cell types; for example, in the case of microglia, if they are predominantly derived from YS tagged progenitors, they should express *eYFP* in the adult CNS when 4′OHT is injected at E7.5 and not at E8.5. In contrast, circulating leukocytes, including monocytes that are known to derive from AGM hematopoietic progenitors, will present the opposite profile, expressing eYFP when 4′OHT is injected at E8.5 instead of E7.5. In addition, if the microglial population does predominantly derive from YS progenitors without a significant contribution from fetal liver- or bone marrow-derived hematopoiesis, they should be tagged at a higher level than circulating leukocytes, which derive predominantly from mature hematopoiesis. To test this hypothesis, we injected the mice with tamoxifen at closely spaced time points of gestation and compared the number of *eYFP*-tagged microglia and circulating monocytes in the mice as adults. Strikingly, the relative number of tagged microglia in mice injected at E7.25 was much greater than of blood monocytes or other circulating leukocytes (Ginhoux et al., 2010).

In contrast, the relative number of tagged microglia in mice injected from E8.0 onwards decreased dramatically to reach undetectable levels as soon as E8.5, while the relative number of *eYFP*^+^ leukocytes, including monocytes, increased progressively in adult blood. This opposing pattern of recombination in microglia compared to circulating leukocytes strongly supports the idea that the major contribution to microglial numbers comes from YS progenitors, and formally excludes the contribution of definitive hematopoiesis. Altogether these results establish that microglia originate from E7.25 Runx1 YS-derived hematopoietic progenitors, with little, if any, contribution from hematopoietic progenitors arising later in embryonic development. Recent studies confirmed our findings (Schulz et al., 2012; Kierdorf et al., 2013). In particular, the latest study from Kierdorf refined the characterization of the YS precursors that give rise to microglia and identified them as early E8 primitive c-kit^+^ erythromyeloid YS precursors which develop into CD45^+^c-kitloCX3CR1^−^ cells before their maturation and migration into the developing brain as CD45^+^c-kit-CX3CR1^+^ cells (Kierdorf et al., 2013).

Altogether, these studies conclusively demonstrated that primitive macrophages are the embryonic source of the steady-state adult microglial population, which was particularly interesting as it implied that microglia not only have a unique functional specialization within the CNS, but also a unique origin, arising from YS progenitors that maintain themselves by proliferating *in situ* throughout adulthood (Figure 1). Beyond the case of microglia, it also provided startling evidence for a broader conclusion, that primitive macrophages are the ultimate source of a functional immune compartment that persists throughout adulthood. However, the case of the microglia seems to be unique, as other fetal macrophage populations in the embryo will mostly be replaced by fetal liver-derived monocytes that seed the tissues later and differentiate into macrophages, as we have recently shown in the case of Langerhans cells (Hoeffel et al., 2012). Lack of differentiation of fetal liver-derived monocytes into microglial progenitors could result from their lack of intrinsic differentiation potential or lack of access to the developing brain. Corroborating the latter hypothesis, the BBB in rodents is starting to be established at approximately E13.5, at the time of fetal liver monocyte release into the blood circulation (Daneman et al., 2010), but after YS-derived macrophages start to invade the neuro-epithelium from E9.5 (Ginhoux and Merad, 2010), possibly restricting the access of fetal liver-derived cells to the embryonic brain (Figure 1).

## Moving toward harnessing microglia to improve human health

It is well established that microglia are intimately involved in the pathology of neurological disease. However, efforts to elucidate the specific roles of microglia, their activation phenotypes and how they can be harnessed to ameliorate disease, are hampered by the lack of access to sufficient numbers of cells for comprehensive *in vitro* studies. Isolation of primary rodent microglia is generally achieved either by cell sorting or stepwise cell culture, both of which are time-consuming and generally yield few cells. While of limited use in rodents, this approach is entirely unfeasible for obtaining human microglia.

Although there has been some success producing microglia-like cells from bone marrow stem cells and circulating monocytes, they are perhaps poor models of “true” microglia as these cell populations do not share the embryonic origin of the vast majority of microglia in the homeostatic brain. Furthermore, their relatively advanced states of differentiation also make them unsuitable for asking questions about the intrinsic and extrinsic regulators of microglial development and specification. In contrast, pluripotent stem cells are already widely used in investigations of embryonic development, have undergone directed differentiation into astrocytes and neuronal subtypes of the CNS, and even been used for cellular transplantation therapies for neurodegenerative diseases (Park et al., 2008a; Kiskinis and Eggan, 2010; Wu and Hochedlinger, 2011; Ben-David et al., 2012). The exciting discovery that terminally-differentiated somatic cells can be reprogrammed into an embryonic-like “induced pluripotent stem cell” (iPSC) has also opened up new possibilities for disease modeling and developing patient-specific therapies (Takahashi and Yamanaka, 2006; Okita et al., 2007; Takahashi et al., 2007; Wernig et al., 2007; Yu et al., 2007; Park et al., 2008b).

Both embryonic stem cells (ESCs) and iPSCs have virtually unlimited expansion potential, can be cultured under defined conditions to ensure reproducible and scalable differentiation protocols, and are amenable to genetic manipulation to create tools for deeper functional studies. Large-scale *in vitro* generation of microglia would also allow us to perform high-throughput genetic screens aiming to uncover key transcription factors responsible for specifying the microglial phenotype. Even more attractively, we could potentially differentiate iPSCs from human patients to re-create a “disease in a dish,” forming a crucial bridge between animal models (which are often deficient) and pathological disease states in humans. Thus far, efforts to recapitulate neurological disease features *in vitro* from human iPSCs have mainly focused on the afflicted neurons (Dimos et al., 2008; Park et al., 2008b; Lee et al., 2009; Soldner et al., 2009; Marchetto et al., 2010). The development of more complex disease models incorporating multiple cell types, particularly microglia, remains a challenge.

**Figure 2:**
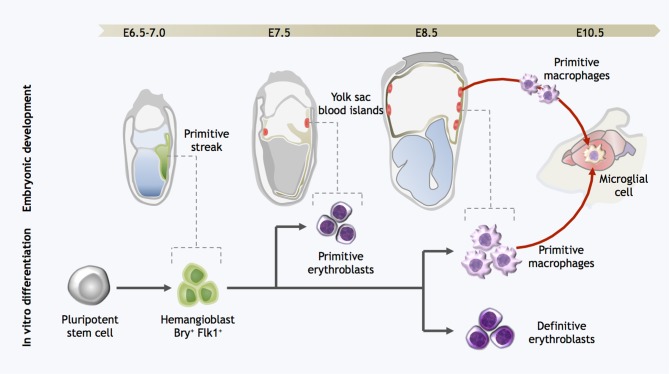
**Strategy for directed differentiation of microglial precursors from pluripotent stem cells.** Microglial differentiation *in vitro* can be achieved by recapitulating the steps of yolk sac hematopoiesis. Hemangioblast cells arise in the posterior primitive streak and migrate into the yolk sac, giving rise to the blood islands. Primitive erythroblasts are observed from E7.0–7.5, followed by definitive erythroblasts and primitive macrophages at E8.5. At the onset of circulation, primitive macrophages exit the yolk sac and seed the developing brain, forming microglia. Likewise, pluripotent stem cells can be differentiated into Bry^+^Flk^+^ cells with hemangioblast properties; these cells are then further differentiated into both primitive and definitive eryothroblasts and primitive macrophages. *In vitro*-derived primitive macrophages may be the functional equivalent to primitive microglia in the embryo.

In contrast to the numerous protocols for efficient generation of astrocytes and neurons from pluripotent stem cells (Lee et al., 2000; Zhang et al., 2001; Hu et al., 2010), there is little literature reporting methods for obtaining phenotypically-correct microglia from the same cell sources. An early study on the differentiation of mouse ESCs (mESCs) into CNS cells in retinoic acid-induced embryoid bodies (EBs) showed that incidental cells expressing microglial markers were generated, in addition to neurons and astrocytes (Angelov et al., 1998). This preliminary success was the motivation for subsequent attempts to generate microglia from mESCs via neuronal differentiation strategies (Tsuchiya et al., 2005; Napoli et al., 2009). In brief, mESCs were induced to differentiate as EBs following withdrawal of leukemia inhibitory factor (LIF), and then progenitors were expanded and further differentiated with neuronal-supportive media and cytokine stimulation. After 21–50 days CD45^low^/CD11b^+^ putative microglia-like cells were observed in these cultures; they expressed surface markers consistent with primary microglia, responded to classical immune activators such as lipopolysaccharide and interferon-γ, and appeared to survive implantation into the mouse brain. However, the low yields and prolonged culture period required, together with our current understanding of the ontogeny of microglia, suggest that these microglia-like cells perhaps are arising as a side population in the neuro-ectodermal differentiation process. On the other hand, we should not discount the possibility that neural cells present in these cultures might have provided a signaling milieu supportive of genuine microglial maturation. For example, brain- and bone marrow-derived Mac-1^+^ progenitors co-cultured on a supportive layer of astroglial cells proliferated and matured into microglial-like cells (Alliot et al., 1991). Sievers and colleagues also showed that co-culture with astrocytes induced blood monocytes and spleen macrophages to adopt a ramified morphology akin to microglia (Sievers et al., 1994a,b). In light of our current understanding that adult microglia originate as primitive macrophages from the embryonic YS, a more effective strategy for approaching pluripotent stem cell differentiation may be to attempt to recapitulate YS hematopoiesis *in vitro* (Figure 2). In the mouse, hemangioblast precursors migrate from the posterior primitive streak into the YS proper, where they form the blood islands and surrounding endothelial cells (Huber et al., 2004). YS hematopoiesis yields primitive erythroblasts as early as E7.0, followed by definitive erythroblasts and macrophage progenitors between E8.5–9.0 (Palis et al., 1999). However, it has been known for some time that these developmental stages can be closely mirrored in mESC differentiation *in vitro*, specifically in terms of the kinetics of hematopoietic gene expression, as well as the order in which hematopoietic progenitors appear. In two modalities of differentiation, either in a co-culture with a hematopoietic-supportive stromal cell layer such as OP9 cells, or as EBs, mESCs sequentially generate *in vitro* equivalents of the primitive streak, hemangioblast, and YS hematopoietic progenitors (Risau et al., 1988; Wiles and Keller, 1991; Nakano et al., 1996; Ogawa et al., 1999; Kennedy and Keller, 2003; Hirai et al., 2005). These processes in mESCs are controlled by the same molecular regulators as those operative during early hematopoiesis *in vivo*. For example, mouse embryos with targeted gene disruption of the hematopoietic master regulator SCL/Tal-1 have no YS hematopoiesis and fail to develop beyond E9.5 (Robb et al., 1995; Shivdasani et al., 1995); likewise abrogation of SCL expression in both mouse and human ESCs completely represses early hematopoietic specification (D'souza et al., 2005; Real et al., 2012). Similarly, PU.1 KO ESCs could not be differentiated into macrophages (Henkel et al., 1996; Anderson et al., 1998), consistent with the finding that PU.1 KO embryos lack both macrophages and microglia (McKercher et al., 1996).

However, useful the mouse developmental model may be, the paucity of equivalent human *in vivo* models makes it imperative to develop accurate *in vitro* alternatives using human pluripotent stem cells. Classical lineage tracing studies of blood development in human embryos showed that many essential features of YS hematopoiesis are conserved from mice to humans (Migliaccio et al., 1986; Huyhn et al., 1995; Palis and Yoder, 2001). As was the case in the mouse system, human pluripotent stem cells could also be differentiated into cells representative of the early hematopoietic developmental stages, albeit with extended kinetics (Wang et al., 2004; Zambidis et al., 2005; Kennedy et al., 2007), suggesting that hematopoietic cell emergence in the YS might be closely modeled by human ESC differentiation (Zambidis et al., 2005).

Given their direct ontogenetic relationship, we postulate that techniques for directing primitive macrophage fate specification from pluripotent stem cells will also yield microglial precursors. Our proposed approach is to further refine protocols for recapitulating YS hematopoiesis, with the aim of increasing the yield of primitive macrophages and levels of reproducibility. Serum-based protocols introduce intrinsic variability, so we will turn to a defined serum-free and feeder cell-free procedure, using only specific combinations of cytokines to replicate the developmental signals during embryogenesis. The key challenge in this approach will be to screen appropriate combinations of factors as well as the time window for treatment. Candidate primitive macrophage/microglial cells can then be isolated based on surface marker expression, and assayed for microglial-appropriate phenotypes such as response to immune stimulation, morphological analysis and phagocytic ability (Giulian and Baker, 1986; Sedgwick et al., 1991). Eventually, the ability to engraft within the brain, with classic resting microglial morphology will be the true test of successful differentiation.

## Conclusion

The “origin of murine microglia controversy” is now resolved in steady state conditions and in a few mouse models of CNS pathologies. We have learned that microglia arise from YS macrophages that seed the brain rudiment from the cephalic mesenchyme very early during development, as predicted earlier by the founder of the microglia field, Pio del Rio-Hortega. Importantly, embryonically-derived microglia will maintain themselves until adulthood. While much progress has been made in terms of our understanding of both the origin and importance of microglia, many questions remain unanswered.

As discussed before, this knowledge may have implications for the use of embryonically-derived microglial progenitors in the treatment of brain inflammatory diseases. Moreover, these results have fundamental implications for the understanding of microglial function in CNS development. First, the conservation of primitive macrophages and their YS derivation throughout evolution and across diverse species suggests that microglia play an important physiological role in the development of the CNS. Furthermore, microglial cells are present in all stages of brain development, including the early prenatal stages of neuronal circuit building as well as the post-natal stage of synapse elimination.

An earlier report had highlighted that, in contrast to their broad distribution in the adult brain, embryonic microglia have a strikingly uneven distribution during embryogenesis (Perry et al., 1985). Microglial cells accumulate in hot spots that were initially proposed to be most likely related to the clearance of apoptotic bodies and the remodeling of brain tissues. In light of the recent work which has shown that microglia contribute to the control of synaptic connections, it will be interesting to verify whether such hotspots co-localize with areas crucial for neuronal development. This will indicate that microglia play an important role in development of neuronal circuits of the brain and proposes more questions to be answered regarding the integrated development of the neural and immune systems. Such questions also have tremendous implications beyond the simple biology of microglia. Do defects affecting microglial development have a long-term impact on the functional vulnerability of CNS? And do defects in microglial function perhaps contribute to synaptic abnormalities seen in some neurodevelopmental disorders? In support of such hypotheses, prenatal inflammation, which triggers the activation of microglia, is thought to be a risk factor for the development of neuropsychiatric disorders such as schizophrenia and autism spectrum disorders in the unborn child (Patterson, 2009, 2011).

Importantly, embryonic microglia will maintain themselves until adulthood via local proliferation during late gestation and post-natal development as well as in the injured adult brain in reaction to inflammation. They are unlikely to be replaced by blood-derived monocytes or any bone marrow-derived cells. Nevertheless, during certain inflammatory conditions found for example after bone marrow transplantation or in chronic neurodegenerative diseases such as Multiple Sclerosis and Alzheimer's disease (Simard and Rivest, 2006; Jung and Schwartz, 2012), the recruitment of monocytes or other bone marrow-derived progenitors can supplement the microglial population to some extent, but we do not understand whether these cells persist and become integrated, or are a temporary addition to the endogenous population. The interactive dynamic between embryonic and adult microglial populations requires further study: We need now to understand to what extent the endogenous microglia is replaced, where and how it is done, and if engrafted cells have a selective advantage over the endogenous microglia, how well they “compete” against the endogenous embryonic microglial population and how long they will persist. Finally, we do not know yet precisely if these bone marrow-derived microglia can fulfill the functional roles of the endogenous population.

### Conflict of interest statement

The authors declare that the research was conducted in the absence of any commercial or financial relationships that could be construed as a potential conflict of interest.
